# Complete genome analysis of *Bacillus subtilis* derived from yaks and its probiotic characteristics

**DOI:** 10.3389/fvets.2022.1099150

**Published:** 2023-01-11

**Authors:** Aoyun Li, Meng Wang, Yu Zhang, Zhengrong Lin, Mengen Xu, Lei Wang, Muhammad Fakhar-e-Alam Kulyar, Jiakui Li

**Affiliations:** ^1^College of Veterinary Medicine, Huazhong Agricultural University, Wuhan, China; ^2^College of Animal Science, Wenzhou Vocational College of Science and Technology, Wenzhou, China; ^3^Department of Animal Nutrition and Feed Science, College of Animal Science and Technology, Huazhong Agricultural University, Wuhan, China; ^4^College of Animals Husbandry and Veterinary Medicine, Tibet Agricultural and Animal Husbandry University, Linzhi, China

**Keywords:** yak, probiotics, *Bacillus subtilis*, genome, KEGG

## Abstract

Probiotics have attracted attention due to their multiple health benefits to the host. Yaks inhabiting the Tibetan plateau exhibit excellent disease resistance and tolerance, which may be associated with their inner probiotics. Currently, research on probiotics mainly focuses on their positive effects on the host, but information regarding their genome remains unclear. To reveal the potential functional genes of *Bacillus subtilis* isolated from yaks, we sequenced its whole genome. Results indicated that the genomic length of *Bacillus subtilis* was 866,044,638 bp, with 4,429 coding genes. The genome of this bacteria was composed of one chromosome and one plasmid with lengths of 4,214,774 and 54,527 bp, respectively. Moreover, *Bacillus subtilis* contained 86 tRNAs, 27 rRNAs (9 16S_rRNA, 9 23S_rRNA, and 9 5S_rRNA), and 114 other ncRNA. KEGG annotation indicated that most genes in *Bacillus subtilis* were associated with biosynthesis of amino acids, carbon metabolism, purine metabolism, pyrimidine metabolism, and ABC transporters. GO annotation demonstrated that most genes in *Bacillus subtilis* were related to nucleic acid binding transcription factor activity, transporter activity, antioxidant activity, and biological adhesion. EggNOG uncovered that most genes in *Bacillus subtilis* were related to energy production and conversion, amino acid transport and metabolism, carbohydrate transport and metabolism. CAZy annotation found glycoside hydrolases (33.65%), glycosyl transferases (22.11%), polysaccharide lyases (3.84%), carbohydrate esterases (14.42%), auxiliary activities (3.36%), and carbohydrate-binding modules (22.59%). In conclusion, this study investigated the genome and genetic properties of *Bacillus subtilis* derived from yaks, which contributed to understanding the potential prebiotic mechanism of probiotics from the genetic perspective.

## Introduction

Yaks (*Bos grunniens*) are an ancient breed of the high-elevation mionectic environment of Tibetan plateau (3,000 above sea level) characterized by cold-resistance and strong anti-adversity (1–3). Early investigations showed that yaks have settled in the Qinghai-Tibet Plateau for thousands of years, which exhibited excellent adaptability to the harsh local environment (3, 4). Statistically, the population of yaks in China has reached 16 million, accounting for more than 90% of the world's total population (5, 6). Yaks are the important source of milk, meat and even vehicle for local herders, which plays an important roles in the agricultural and economic development of the Qinghai-Tibet Plateau (7, 8). Therefore, any disease appeared in the yaks may cause huge economical losses to the herdsmen on the cold plateau. However, the yaks especially in juvenile populations prone to gastrointestinal diseases because of the nutritional deficiency and harsh environment (9, 10). Increasing evidence indicated that environmental factors such as hypothermia and hypoxia could affect the evolution of species (11, 12). For instance, the gut microbiota was significantly different between human inhabiting Tibet Plateau and inland plain due to differences in living circumstances (12). Moreover, Xin et al. also revealed significant differences in the gut microbiota between yaks and other breeds of cattle (2). Consequently, yaks have evolved a unique gut microbial community structure with the environment.

Gut microbiota is a complicated and dynamic microecosystem that consists of ~ 100 trillion microorganisms involving over 2,000 diverse species (13–15). Notably, some strains in the gut microbiota such as *Bacillus subtilis, Bacillus licheniformis, Pediococcus lactis*, etc. are recognized as probiotics (16–18). Probiotics are regarded as beneficial commensals that provide multiple health benefits to the host involving antibacterial activity, immunological regulation and intestinal barrier improvement (19–21). Moreover, recent publications on probiotics also demonstrated their properties to lower cholesterol, regulate gut microbiota and alleviate liver-related diseases (22–24). For instance, Sun et al. demonstrated the remarkable efficacy of *Bacillus subtilis* alleviated obese by stabilizing the gut microbiota in some exploratory mice studies (25). Likewise, Zhao et al. indicated that *Bacillus subtilis* could alleviate alcoholic liver disease by regulating gut microbiota and intestinal permeability (24). Although probiotics supplementation has been shown to be a promising strategy for alleviating diarrhea, growth retardation and liver-related diseases, studies regarding probiotics in yaks remains scarce.

Recently, high-throughput sequencing technologies, including metagenomes and metabolomes, have been successfully applied to analyze the complex structure of gut microbiota and their metabolites (26–28). In-depth dissection of gut microbial composition and structure is beneficial for understanding the importance and underlying mechanisms of gut microbiota in intestinal function and host health (29, 30). Additionally, high-throughput genomic technology can also conduct a comprehensive analysis of probiotic whole genome sequences, which contribute to exploring potential probiotic mechanisms (31, 32). Previous studies indicated that the genome of *Bacillus subtilis* derived from different sources such as tilapia and soil has been well-characterized and revealed the reason for their probiotic properties (33). However, until now, studies regarding the genome characteristics of *Bacillus subtilis* isolated from yaks remains scarce. Numerous studies indicated that the adaption to the extreme environment of the Tibet plateau may potentially be associated with gut microbiota and adapted microorganism, which may be dramatically affected by the structure of their genomes (1, 2). Consequently, we performed this study to investigate the genome characterization of *Bacillus subtilis* isolated from yaks.

## Materials and methods

### Bacterial strains

The 6 ± 2 yak calves were purchased and raised at free ranged area up to the age of 2 years. Afterwards, the *Bacillus subtilis* was isolated from their intestinal cecum after slaughtering. The *Bacillus subtilis* was inoculated into Luria-Bertani broth at 37°C for 24 h for multiplication culture (34). Subsequently, the bacterial solution was sent to Beijing Biomarker Technologies Co, LTD for whole-genome sequencing.

### Genome DNA extraction

Prior to the bacterial DNA extraction, the bacterial solution needs to be centrifuged at 8,000 rpm for 40 min to obtain the bacterial pellet. Subsequently, the bacterial pellet was washed three times with sterile phosphate buffer as described above. The genomic DNA of bacterial pellet was extracted using the QIAamp DNA Mini Kit (QIAGEN, Hilden, Germany) based on the manufacturer's recommendations. Moreover, the agarose gel electrophoresis and quantitative of extracts were performed to guarantee its integrity, purity and concentration meet the subsequent analysis requirements.

### Library construction and sequencing

BluePippin automatic nucleic acid recovery system was used to recycle and purify targeted DNA fragments. DNA samples were subjected to fragment, damage repair, end repair, magnetic bead purification and adapter ligation. The SQK-LSK109 ligation Sequencing Kit was applied to construct DNA library. Subsequently, the Invitrogen Qubit 3.0 was used for measuring library concentration. The final library was performed sequencing using Illumina HiSeqTM 2000 sequencer.

### Quality control and sequence assembly

The raw data obtained from sequencing needs to be quality controlled to remove low quality and short sequences and obtain available clean data. Subsequently, the statistical analysis of clean data were performed to obtain relevant information such as the total amount of data, the length of reads, and the distribution of quality values. The second- and third-generation data were assembled using Unicycler software and the reads were aligned to the assembled sequences. The distribution of sequencing depth was counted and the assembled sequence was distinguished from chromosomal sequence or plasmid sequence according to the sequence length and alignment, and whether the sequence was circular or not. (1) Preliminary assembly: the SPAdes was used to assemble the second-generation data into a frame diagram; (2) Create bridges: the miniasm and Racon was used to add three generations of data to the frame diagram, and the three generations of long read data was used to create bridges; (3) Bridge application: In the presence of conflict, the Unicycler was used to select a high-scoring bridge according to the quality score corresponding to the created bridge; (4) Looping judgment and final confirmation: the start_genes was used to start site correction and final confirmation, screen chromosome and plasmid sequences and assemble the chromosome sequence into a circular genome.

### Bioinformatics analysis

#### Gene prediction

Coding gene predictions were performed on newly sequenced genomes using GeneMarkS (Version 4.17) software. tRNA, rRNA, and sRNA were predicted by tRNAscan-SE software (Version 1.3.1), rRNAmmer software (Version 1.2), and Rfam database. Interspersed and tandem repeat predictions were performed using the prediction software RepeatMasker (Version open-4.0.5) and TRF (Tandem Repeats Finder, Version 4.07b). IslandPath-DIOMB software (Version 0.2), phispy software (Version 2.3), and CRISPRdigger (Version 1.0) were used to predict gene islands, prophages, and CRISPR prediction on the sample genome, respectively.

#### Functional annotation

Predicted genes were BLAST aligned with various functional databases. Based on the BLAST result of each sequence, the alignment with the highest score is annotated (default identity ≥ 40%, coverage ≥ 40%). The predicted coding genes were annotated in the GO (Gene Ontology), KEGG (Kyoto Encyclopedia of Genes and Genomes), COG (Cluster of Orthologous Groups of proteins), NR (Non-Redundant Protein Database), TCDB (Transporter Classification Database), CAZy (Carbohydrate-Active EnZymes Database) function database database, and the pathogen-host interaction database was annotated. According to the above sequencing and bioinformatics analysis results, the genome circle map of *Bacillus subtilis* was drawn.

## Results

### Data acquisition and analysis

**Figure 1:**
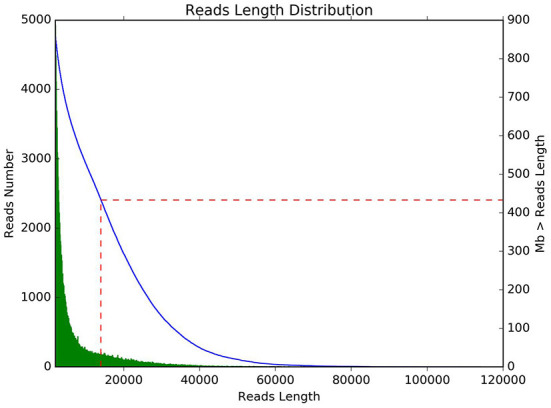
The length distribution of sequencing. The horizontal axis represents reads length, and the vertical axis represents reads number.

Quality control statistics for *Bacillus subtilis* are shown in Table 1. In this research, a total of 109,397 reads and 866,044,638 bases were obtained. The average length of the reads was 168,691 bp and the maximum length of the reads was 114,102 bp. The sequencing reads N50 length and N90 length were 14,073 and 3,043 bp, respectively. The average sequencing quality of reads was 11.12. Subsequently, statistical analysis of the length and data volume of the reads was performed and found that the reads were mainly concentrated in 10,000–20,000 bp (Figure 1).

**Table 1:** The information of quality control of *Bacillus subtilis*.

**Reads type**	**SeqNum**	**SumBase**	**N50Len**	**N90Len**	**MeanLen**	**MaxLen**	**MeanQual**
Clean reads	109,397	866,044,638	14,073	3,043	7,916	114,102	11.12

### Basic characteristics of genome

**Figure 2:**
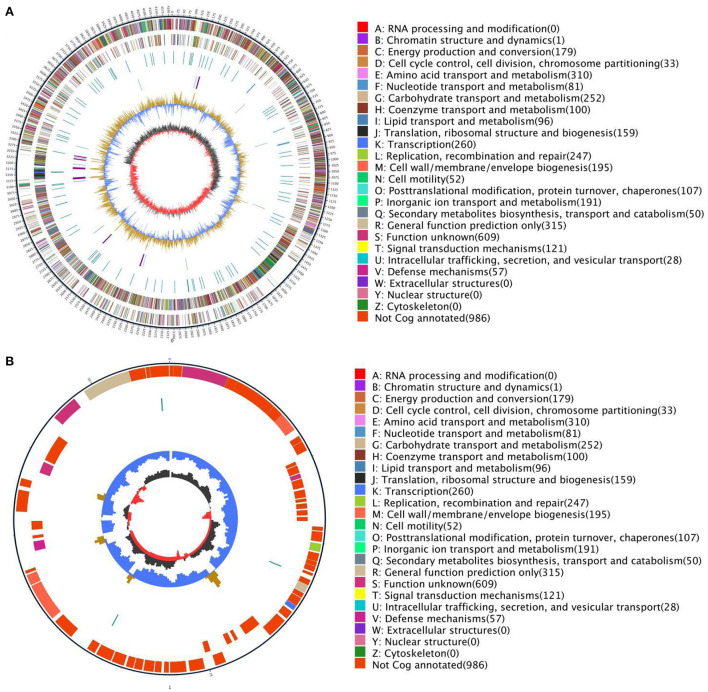
Circular genome map of *Bacillus subtilis* **(A)**. The genome circular maps of *Bacillus subtilis* plasmid **(B)**. The outermost circle is the mark of the genome size and each scale is 5 kb; the second and third circles are the genes on the positive and negative strands of the genome respectively, and different colors represent different COG functional classifications; the fourth circle is the repetitive sequence; the fifth circle is tRNA and rRNA, the blue is tRNA, and the purple is rRNA; the sixth circle is the GC content. The light yellow part indicates that the GC content in this area is higher than the average GC content of the genome. The higher the peak value, the greater the difference from the average GC content. The blue part indicates that the GC content of this region is lower than the average GC content of the genome; the innermost circle is GC-skew, the dark gray represents the area where the G content > C, and the red represents the area where the C content > G.

The basic information of the genome of *Bacillus subtilis* is shown in Figure 2. The genome of *Bacillus subtilis* consists of 1 chromosome and 1 plasmid and the chromosome and plasmid genome are circular. The length of chromosome and plasmid are 4,214,774 and 54,527 bp, respectively.

### Coding gene and non-coding RNA

**Figure 3:**
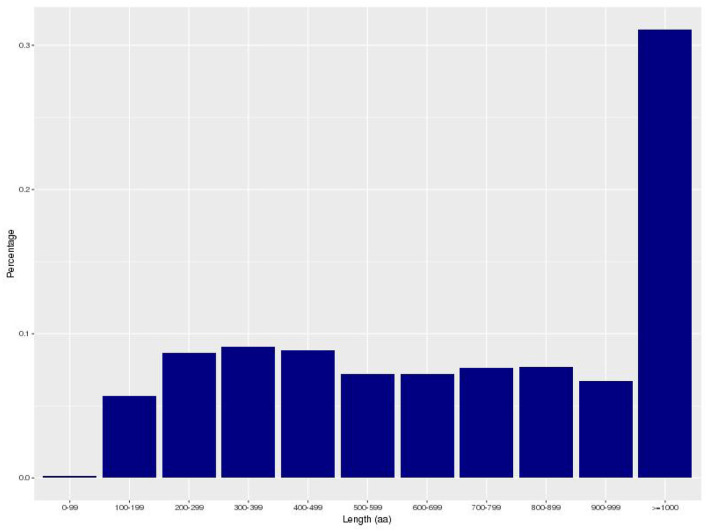
The map of coding gene length distribution. The horizontal axis represents the length of the protein sequence and the vertical axis represents the proportion.

The gene encoding information of *Bacillus subtilis* is shown in Table 2. A total of 4,429 coding genes were predicted, and the total length of all coding genes was 3,751,617 bp. The length of coding genes ranges from 90 to 10,764 bp, with an average length of 847 bp. The majority of genes are between 100 and 999 bp in length (Figure 3). *Bacillus subtilis* contains 86 tRNAs, 27 rRNAs (9 16S_rRNA, 9 23S_rRNA and 9 5S_rRNA), and 114 other ncRNA (Supplementary Table 1).

**Table 2:** The prediction results of coding gene.

**Geneset number**	**Total length (bp)**	**Average (bp)**	**Max length (bp)**	**Min length (bp)**
4,356	3,705,798	850	10,764	90

### Genomic island and prophage prediction

Twenty genomic islands were predicted in the genome of *Bacillus subtilis* (Table 3). The length of the predicted genomic islands are 4,915, 12,686, 9,247, 10,861, 39,818, 40,088, 6,130, 8,870, 24,920, 6,441, 10,096, 11,278, 6,245, 8,489, 18,551, 36,598, 36,110, 17,647, 5,638, and 10,887, respectively. Moreover, the total and mean length of the genomic islands are 325,515 and 16,275, respectively (Supplementary Table 2). A total of 3 prophage were predicted with lengths of 8,848, 84,557, and 134,638, respectively (Table 4). Moreover, the total length and mean length of the predicted prophage are 228,043 and 76,014, respectively.

**Table 3:** Genomic island prediction results statistics.

**Genomic islands**	**Scaffold_id**	**Start**	**End**	**Length (bp)**
Genomic_island_1	Contig00001	327,094	332,008	4,915
Genomic_island_2	Contig00001	357,791	370,476	12,686
Genomic_island_3	Contig00001	696,916	706,162	9,247
Genomic_island_4	Contig00001	708,313	719,173	10,861
Genomic_island_5	Contig00001	724,211	764,028	39,818
Genomic_island_6	Contig00001	1,102,910	1,142,997	40,088
Genomic_island_7	Contig00001	1,254,876	1,261,005	6,130
Genomic_island_8	Contig00001	1,273,090	1,281,959	8,870
Genomic_island_9	Contig00001	1,390,699	1,415,618	24,920
Genomic_island_10	Contig00001	1,438,503	1,444,943	6,441
Genomic_island_11	Contig00001	1,991,961	2,002,056	10,096
Genomic_island_12	Contig00001	2,090,597	2,101,874	11,278
Genomic_island_13	Contig00001	2,146,419	2,152,663	6,245
Genomic_island_14	Contig00001	2,604,742	2,613,230	8,489
Genomic_island_15	Contig00001	2,697,661	2,716,211	18,551
Genomic_island_16	Contig00001	3,053,174	3,089,771	36,598
Genomic_island_17	Contig00001	3,132,900	3,169,009	36,110
Genomic_island_18	Contig00001	3,360,721	3,378,367	17,647
Genomic_island_19	Contig00001	4,053,466	4,059,103	5,638
Genomic_island_20	Contig00001	4,142,648	4,153,534	10,887

**Table 4:** Statistics of prophage prediction results.

**Prophage_id**	**Scaffold_id**	**Start**	**End**	**Length (bp)**
Prophage_1	Contig00001	709,561	718,408	8,848
Prophage_2	Contig00001	1,050,842	1,135,398	84,557
Prophage_3	Contig00001	1,305,581	1,440,218	1,440,218

### CRISPR predictive analysis

A total of 10 CRISPR were predicted and the average spacer length were 19, 19, 19, 45, 19, 38, 19, 24, 55, and 58, respectively (Table 5). The average repeat length of the predicted CRISPR were 21, 21, 21, 21, 38, 22, 22, 19, 26, and 34, respectively.

**Table 5:** Results of CRISPR prediction.

**CRISPR id**	**Contig id**	**Start**	**End**	**RN**	**ARL (bp)**	**SN**	**ASL (bp)**
CRISPR.1	Contig00001	738,657	738,717	2	21	1	19
CRISPR.2	Contig00001	1,126,406	1,126,466	2	21	1	19
CRISPR.3	Contig00001	1,983,791	1,983,851	2	21	1	19
CRISPR.4	Contig00001	2,518,060	2,518,146	2	21	1	45
CRISPR.5	Contig00001	2,518,351	2,518,445	2	38	1	19
CRISPR.6	Contig00001	2,751,179	2,751,440	5	22	4	38
CRISPR.7	Contig00001	2,963,697	2,963,759	2	22	1	19
CRISPR.8	Contig00001	3,805,753	3,806,029	7	19	6	24
CRISPR.9	Contig00001	3,808,304	3,808,572	4	26	3	55
CRISPR.10	Contig00002	32,279	32,404	2	34	1	58

RN, repeat number; ARL, Average repeat length; SN, Spacer number; ASL, average spacer length.

### GO annotation results

**Figure 4:**
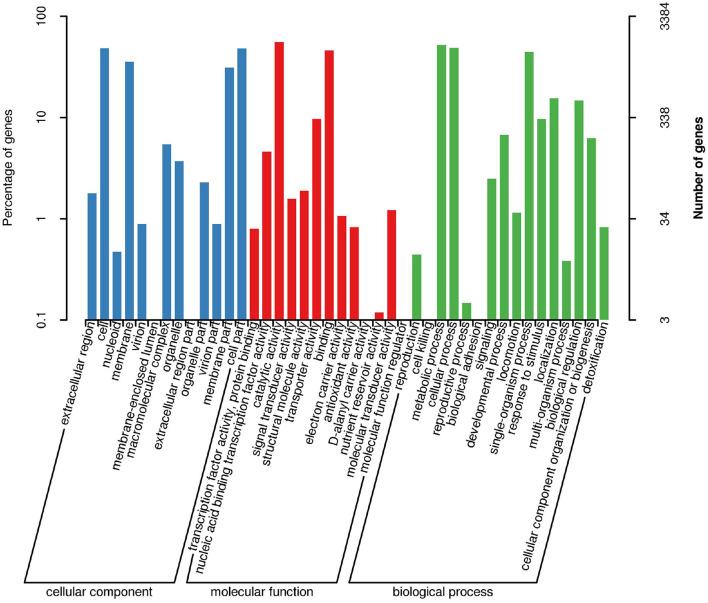
Functional classification results of GO annotation. The horizontal axis represents the content of each category of GO, the left side of the vertical axis represents the percentage of the number of genes, and the right represents the number of genes.

*Bacillus subtilis* was performed for functional prediction using the GO database and the genic functions were divided into three categories, including biological processes, molecular functions, and cellular components (Figure 4). The number of genes involved in biological processes, molecular functions, and cellular components was 100, 200, and 300, respectively. In terms of cellular component, most genes are associated with extracellular region, cell, membrane, macromolecular complex, organelle, organelle part, virion part, membrane part, and cell part. In terms of molecular function, most genes are associated with transcription factor activity, protein binding, nucleic acid binding transcription factor activity, catalytic activity, signal transducer activity, structural molecule activity, transporter activity, binding, electron carrier activity, antioxidant activity, molecular transducer activity, and molecular function regulator. In terms of biological process, most genes are related to cell killing, metabolic process, cellular process, reproductive process, biological adhesion, signaling, developmental process, locomotion, single-organism process, response to stimulus, localization, multi-organism process, biological regulation, cellular component organization or biogenesis, and detoxification.

### KEGG annotation results

**Figure 5:**
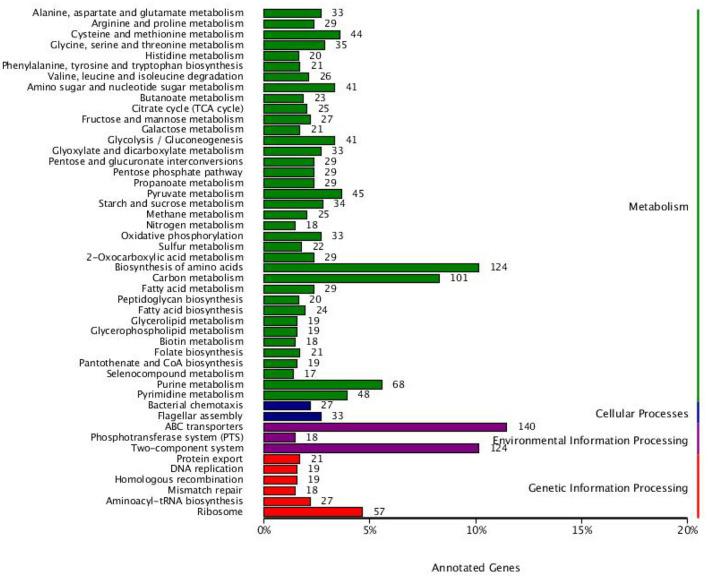
Functional classification results of KEGG annotation. The histogram displays the number of KEGG pathway genes involved and the colors signify various systems.

In terms of metabolism, most genes are related to alanine, aspartate and glutamate metabolism, arginine and proline metabolism, cysteine and methionine metabolism, glycine, serine and threonine metabolism, histidine metabolism, phenylalanine, tyrosine and tryptophan biosynthesis, valine, leucine and isoleucine degradation, amino sugar and nucleotide sugar metabolism, butanoate metabolism, citrate cycle (TCA cycle), fructose and mannose metabolism, galactose metabolism, glycolysis/gluconeogenesis, glyoxylate and dicarboxylate metabolism, pentose and glucuronate interconversions, pentose phosphate pathway, propanoate metabolism, pyruvate metabolism, starch and sucrose metabolism, methane metabolism, nitrogen metabolism, oxidative phosphorylation, sulfur metabolism, 2-Oxocarboxylic acid metabolism, biosynthesis of amino acids, carbon metabolism, fatty acid biosynthesis, peptidoglycan biosynthesis, fatty acid metabolism, glycerolipid metabolism, glycerophospholipid metabolism, biotin metabolism, folate biosynthesis, pantothenate and CoA biosynthesis, selenocompound metabolism, purine metabolism, and pyrimidine metabolism (Figure 5). In terms of cellular processes, most genes are related to bacterial chemotaxis and flagellar assembly. In terms of environmental information processing, most genes are related to ABC transporters, phosphotransferase system (PTS) and two-component system. In terms of genetic information processing, most genes are related to protein export, DNA replication, homologous recombination, mismatch repair, aminoacyl-tRNA biosynthesis, and ribosome.

### EggNOG annotation results

**Figure 6:**
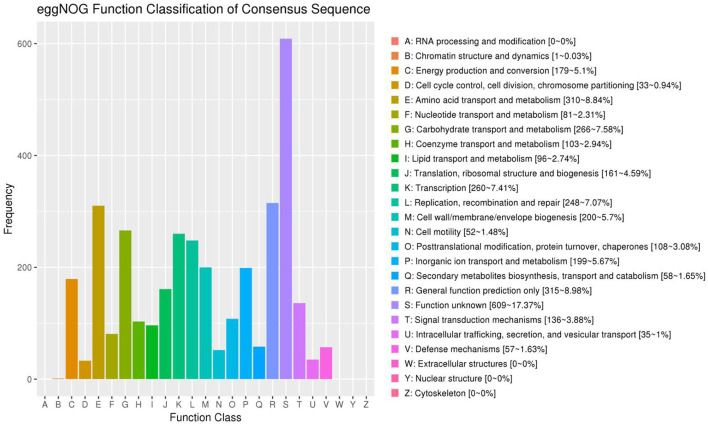
Functional classification results of eggNOG annotation. The horizontal axis represents the content of each category of eggNOG, and the vertical axis represents the relative proportion of the number of corresponding functional genes.

EggNOG annotation results indicated that most genes are related to chromatin structure and dynamics (1–0.03%), energy production and conversion (179–5.1%), cell cycle control, cell division, chromosome partitioning (33–0.94%), amino acid transport and metabolism (310–8.84%), nucleotide transport and metabolism (81–2.31%), carbohydrate transport and metabolism (266–7.58%), coenzyme transport and metabolism (103–2.94%), lipid transport and metabolism (96–2.74%), translation, ribosomal structure and biogenesis (161–4.59%), transcription (260–7.41%), replication, recombination and repair (248–7.07%), cell wall/membrane/envelope biogenesis (200–5.7%), cell motility (52–1.48%), post-translational modification, protein turnover, chaperones (108–3.08%), inorganic ion transport and metabolism (199–5.67%), secondary metabolites biosynthesis, transport and catabolism (58–1.65%), general function prediction only (315–8.98%), function unknown (609–17.37%), signal transduction mechanisms (136–3.88%), intracellular trafficking, secretion, and vesicular transport (35–1%), and defense mechanisms (57–1.63%; Figure 6).

### CAZy database annotation result

**Figure 7:**
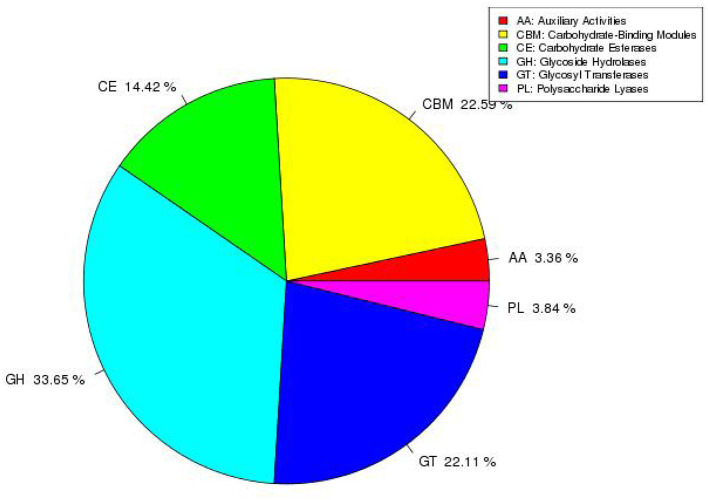
Distribution and scale diagram of carbohydrase.

The CAZy database annotation results are shown in Figure 7. The results show that a total of six functions are annotated such as glycoside hydrolases (GH), glycosyl transferases (GT), polysaccharide lyases (PL), carbohydrate esterases (CE), auxiliary activities (AA), and carbohydrate-binding modules (CBM). The proportion of annotated functions are 33.65, 22.11, 3.84, 14.42, 3.36, and 22.59%, respectively. Moreover, the number of genes involved in the annotated functions are 70, 46, 8, 30, 7, and 47, respectively.

## Discussion

Typically, yaks are mainly free-range on the Qinghai-Tibet Plateau and not supplemented with additional nutrients, which causes their slow growth and high disease incidence (35, 36). Additionally, juvenile yaks exhibit high rates of diarrhea because of the harsh environment, nutrient deprivation and immature gut microbial community, resulting in massive mortality and economic losses (9). Dietary intervention has long been regarded as a vital way to decrease morbidity rate and increase production performance, which has attracted growing attention (37, 38). As a recognized probiotic, *Bacillus subtilis* is widely applied in animal husbandry due to its strong stress resistance and positive effects on the host (39, 40). For instance, Zhang et al. demonstrated that *Bacillus subtilis* PB6 administration dramatically increased villus height and maintain intestinal mucosal barrier function of ducks (41). Analogously, Mohamed et al. found that *Bacillus subtilis* administration in the basal diet could dramatically enhance the growth performance of broiler chickens by regulating gut microbiota and intestinal morphology (42). However, most of the present studies focus on the beneficial mechanism of probiotics on the host and few research pay attention to the genomic characteristics of probiotics. Whole genome sequence analysis can more comprehensively evaluate the safety of *Bacillus subtilis* and gradually deepen from traditional phenotypic analysis to molecular mechanism and genetic characteristics (43, 44). To further understand of the genomic bioinformatics characteristics of *Bacillus subtilis* isolated from yaks, this study conducted the whole genome analysis to analyze the genome characteristics and functional predictions based on the previous research on the *in vitro* probiotic characteristics of *Bacillus subtilis*.

Carbohydrate-active enzymes are encoded by the gut microbial genomes of animals, which play a key regulatory role in carbohydrate breakdown. Early investigations indicated that carbohydrate-active enzymes were essential for host nutrient absorption (45). Glycoside hydrolases are an important class of enzymes that usually play a key role in the hydrolysis of glycosidic bonds of carbohydrates. Previous studies indicated that there are hundreds of different glycosyltransferases involved in the biosynthesis of disaccharides, oligosaccharides and polysaccharides, contributing to the formation of glycosidic bonds (46). In this study, the genome of *Bacillus subtilis* contains genes encoding GHs and GTs, and these active enzymes enable *Bacillus subtilis* to synthesize carbohydrates and metabolize sugars.

Amino acids are the basic constituent unit of proteins, which play key roles in energy conversion, protein biosynthesis and protein biosynthesis (47). For instance, arginine can participate in the ornithine cycle and convert the ammonia produced in the body into non-toxic urea through the ornithine cycle, thereby decreasing the blood ammonia concentration (48). Serine has been demonstrate to possess several metabolic functions including providing precursors for the synthesis of phosphoglycerides, neurotransmitters, sphingolipids, proteins and phosphatidylserine (49, 50). Tryptophan can participate in the renewal of plasma proteins in animals, promote the role of riboflavin and the synthesis of niacin and heme, which contributes to increasing the antibodies in the fetuses of pregnant animals and promote lactation in dairy cows and sows (51). Early surveys indicated that tryptophan deficiency caused growth arrest, weight loss, fat accumulation and testicular atrophy in breeding stock (52). Previous studies have shown that phenylalanine and tyrosine could synthesize important neurotransmitters and hormones involved in the body's glucose metabolism and fat metabolism (53). Numerous investigations indicated that gut microbiota played key roles in the metabolism and synthesis of amino acids (54). For instance, C*lostridium* in the intestine are key drivers of amino acid fermentation, whereas *Streptococcus* play a key role in the utilization of glutamate and tryptophan. In this study, we annotated a large number of genes related to amino acid metabolism in the genome of *Bacillus subtilis*, such as alanine, aspartate and glutamate metabolism, arginine and proline metabolism, cysteine and methionine metabolism, glycine, serine and threonine metabolism, histidine metabolism, etc., which may be one of the reasons for gut microbiota or probiotics regulate amino acid metabolism. Research on ABC transporters has demonstrate their essential roles in the transport of inorganic ions, monosaccharides, glycans, cholesterol, phospholipids, amino acids, peptides, proteins, toxins, drugs, antibiotics and heterologous substances, which are essential for cellular detoxification, membrane homeostasis, and lipid transport (55). Fatty acids exhibit multiple vital functions such as providing energy, maintaining the relative fluidity of cell membranes and normal physiological functions of cells, esterifying cholesterol to reduce blood cholesterol and triglyceride levels, improving brain cell activity, and enhancing memory (56). Notably, some members of fatty acids such as short-chain fatty acids (SCFAs: acetate, propionate, and butyrate) have attracted increasing attention due to their important roles in host health and metabolism (57). Early surveys on SCFAs provided evidence that they could regulate intestinal pH, intestinal permeability, gut microbiota and attenuate intestinal inflammation (58). Additionally, SCFAs have also been shown to play vital roles in cell proliferation, immune system and preventing intestinal leakage (59, 60). In this study, the genome of *Bacillus subtilis* contains genes encoding ABC transporters, fatty acid metabolism and fatty acid biosynthesis and this may be one of the reasons for probiotics exert beneficial effects on the host.

Biotin, also known as vitamin B7, can enhance the host immune response, infection resistance, maintain normal growth, and development (61, 62). Biotin can be used to treat diseases related to arteriosclerosis, stroke, lipid metabolism disorders, hypertension, coronary heart disease, and blood circulation disorders (63). Conversely, biotin deficiency can result in reproductive decline, skeletal dysplasia, stunted embryonic, and young child growth (63). Folic acid has been demonstrated to promote the formation of red blood cells and play important roles in protein synthesis, cell division, and growth (64, 65). Folic acid deficiency can cause megaloblastic anemia and affect hemoglobin production and cell maturation in red blood cells (65, 66). Currently, folic acid is widely used in animal husbandry due to various important physiological functions. For instance, folic acid administration in the basal diet significantly increase the serum immunoglobulin content, reduce the serum cholesterol and improve the apparent digestibility of dry matter, crude protein, and crude fat of chickens (67, 68). Moreover, folic acid supplementation can also improve the growth performance of piglets (69). In this study, we found that *Bacillus subtilis* contains genes related to folate biosynthesis and biotin metabolism, which is consistent with previous evidence that *Bacillus subtilis* can produce vitamins.

Oxidative stress was previously demonstrate to cause many chronic diseases such as aging, enteritis, atherosclerosis and even cancer (70–72). Moreover, oxidative stress can also decrease meat quality and fertility, seriously threatening livestock production (73, 74). Thus, decreasing oxidative stress is vital for ensuring human and animal health. Probiotics have been widely demonstrated to improve the host's antioxidant capacity by increasing the activity of antioxidant enzymes or activating antioxidant-related pathways (24, 75). Meanwhile, probiotics also have their own antioxidant systems. In this study, we observed the presence of antioxidant activity-related genes in *Bacillus subtilis*, which also validated the antioxidant properties of *Bacillus subtilis*.

In summary, this study first characterized the genome and genetic properties of *Bacillus subtilis* isolated from yaks. Results indicated that the genome of *Bacillus subtilis* contained genes related to anti-oxidation and biological adhesion. Moreover, we performed functional prediction on the genome and found that some genes were involved in amino acid metabolism, vitamin synthesis and metabolism, fatty acid metabolism, etc., which further confirmed that *Bacillus subtilis* isolated from yaks has good probiotic properties. This study revealed beneficial properties of *Bacillus subtilis* from the genomic perspective, which contributed to improving public awareness of *Bacillus subtilis* and providing a theoretical basis for the developing probiotic products. However, this study also has some limitations such as lack of comparative analysis with other bacterial genomes.

## Data availability statement

The datasets presented in this study can be found in online repositories. The names of the repository/repositories and accession number(s) can be found in the article/Supplementary material.

## Ethics statement

The animal study was reviewed and approved by Ethics Committee of Huazhong Agricultural University.

## Author contributions

AL and JL conceived and designed the experiments. YZ, ZL, MX, and LW contributed sample collection and reagents preparation. AL analyzed the data and wrote the manuscript. MW and MK revised the manuscript. All authors reviewed the manuscript. All authors contributed to the article and approved the submitted version.
