# Supplementary material for: Complete genome analysis of Bacillus subtilis derived from yaks and its probiotic characteristics
Source: Front Vet Sci. 2023 Jan 11;9:1099150. doi: 10.3389/fvets.2022.1099150 (PMC9875379; doi:10.3389/fvets.2022.1099150)
Supplement: Supplementary file 1 [file Table_1.docx]

**Supplementary Table S1.** Statistics of noncoding RNA prediction results.

| RNA classify | Number |
| --- | --- |
| 16S_rRNA  23S_rRNA  5S_rRNA  tRNA  other ncRNA | 9  9  9  86  114 |
