# Supplementary material for: Complete genome analysis of Bacillus subtilis derived from yaks and its probiotic characteristics
Source: Front Vet Sci. 2023 Jan 11;9:1099150. doi: 10.3389/fvets.2022.1099150 (PMC9875379; doi:10.3389/fvets.2022.1099150)
Supplement: Supplementary file 2 [file Table_2.docx]

**Supplementary Table S2.** The information of genomic island prediction.

| genomic islands number | genomic islands total length | genomic islands mean length |
| --- | --- | --- |
| 20 | 325,515 | 16,275 |
